# Allergic contact cheilitis caused by propolis: case report

**DOI:** 10.31744/einstein_journal/2022RC6151

**Published:** 2022-02-02

**Authors:** Paulo Eduardo Silva Belluco, Rosana Zabulon Feijó Belluco, Carmelia Matos Santiago Reis

**Affiliations:** 1 Escola Superior de Ciências da Saúde Brasília DF Brazil Escola Superior de Ciências da Saúde, Brasília, DF, Brazil.

**Keywords:** Dermatitis, contact, Cheilitis, Propolis, Patch tests

## Abstract

Propolis is a lipophilic resin extracted from plants by bees. The purpose of this case report was to show the importance of this substance as cause of allergic contact cheilitis. A 21-year-old female patient complained of pruritic perioral eczema for 5 years. In the past months it also affected the neck. After diagnosing contact dermatitis, she was submitted to a patch test with a Latin American baseline series. The result was strongly positive for propolis (++) and weakly positive for perfume mix I (+). After the test, the patient revealed she had been using propolis drops, *per oris*, for 10 years. The worsening of the condition was due to increased dose, aiming “to improve immunity”, during the coronavirus disease 2019 (COVID-19) pandemic. The contact allergy to propolis might be increasing due to the widespread use of natural products. Propolis is a sensitizer to be considered in patients with long-lasting cheilitis.

## INTRODUCTION

Contact allergic dermatitis can occur in different anatomical regions, including the perioral region. The lips are often exposed to cosmetic products. Patients may sometimes decide to use natural products, and believe they are free from irritants or allergens.^(1)^

Cheilitis is a common presentation of propolis allergy.^(2)^ The purpose of this report is to show the case of a young woman who presented with allergic contact cheilitis for 5 years. Under the pretext of improving immunity due to the coronavirus disease 2019 (COVID-19) pandemic, the patient increased the dose of propolis she had been using for years, causing the eczema to involve the neck, leading led her to seek medical assistance. In addition, the importance of diagnosing propolis-related contact allergy is emphasized to the medical community.

## CASE REPORT

**Figure 1:**
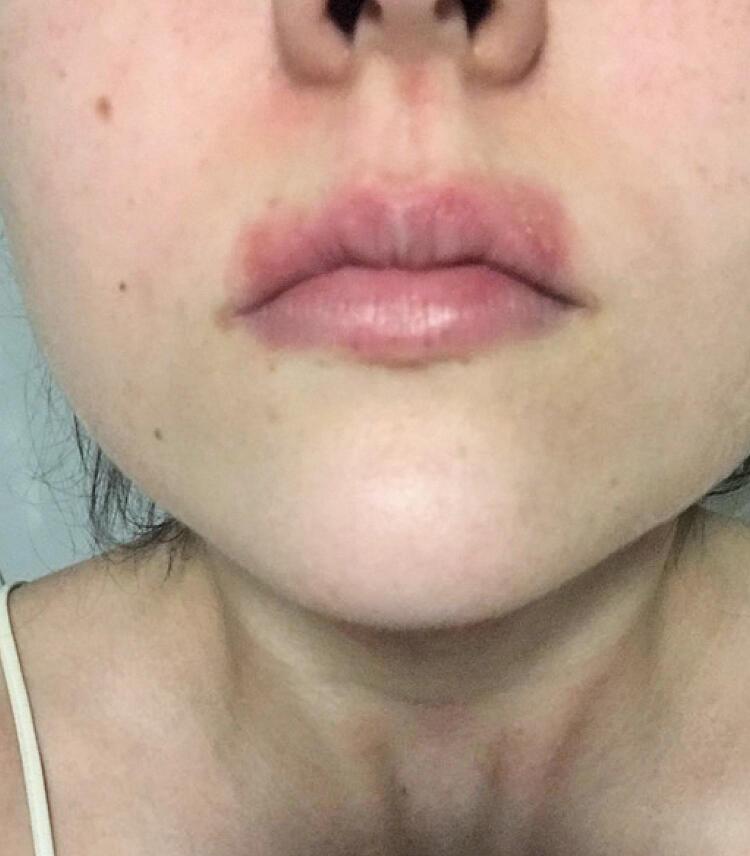
Erythematous perioral plaque with vesicles

A 21-year-old female student reported she has had perioral eczema for 5 years, characterized by an erythematous plaque adjacent to the lips, with cracks and peeling, accompanied by itching and local pain (Figure 1).

**Figure 2:**
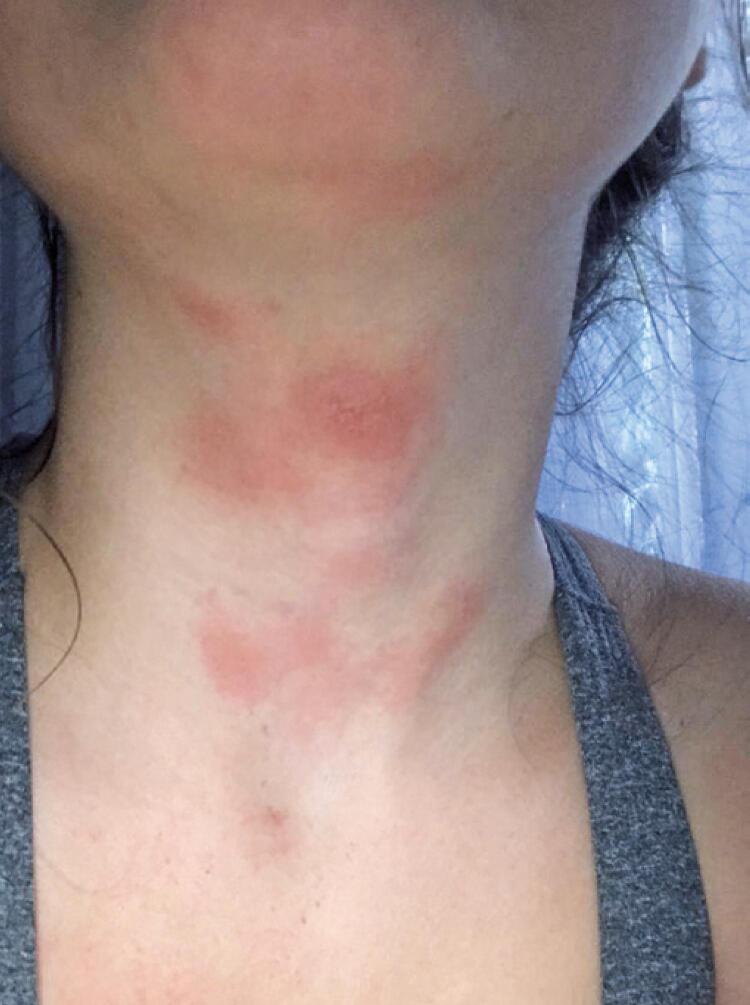
Sparse erythematous plaques on anterior aspect of neck

The clinical picture was continuous, although she noticed there were periods of calmness and exacerbations, unrelated to triggering factors. Four months ago, at the beginning of the COVID-19 pandemic, the patient noticed a marked worsening, with the appearance of an intensely pruritic eczema on the anterior aspect of her neck, which made her seek medical assistance to solve the problem (Figure 2).

She denied any relation to food or the use of any cosmetics. She specifically reported not using lipstick. She only used dexpanthenol-based moisturizing lip cream, without knowing for sure if this was an improvement or worsening factor. She also denied using any other medication. With a past history of allergic rhinitis, she had undergone allergy-specific immunotherapy; however, she was currently asymptomatic only with control of home environment for dust and dust mites. The family history included her brother, who had already suffered anaphylaxis from an ant bite (allergy to hymenoptera).

The diagnostic evaluation took into consideration the fact she was an atopic patient. Thus, skin test and specific immunoglobulin E dosage for food and inhalants were performed, and were positive only for *Dermatophagoides farinae* and *Dermatophagoides pteronyssinus*, which was considered related to controlled rhinitis, but not associated with the skin condition.

**Figure 3:**
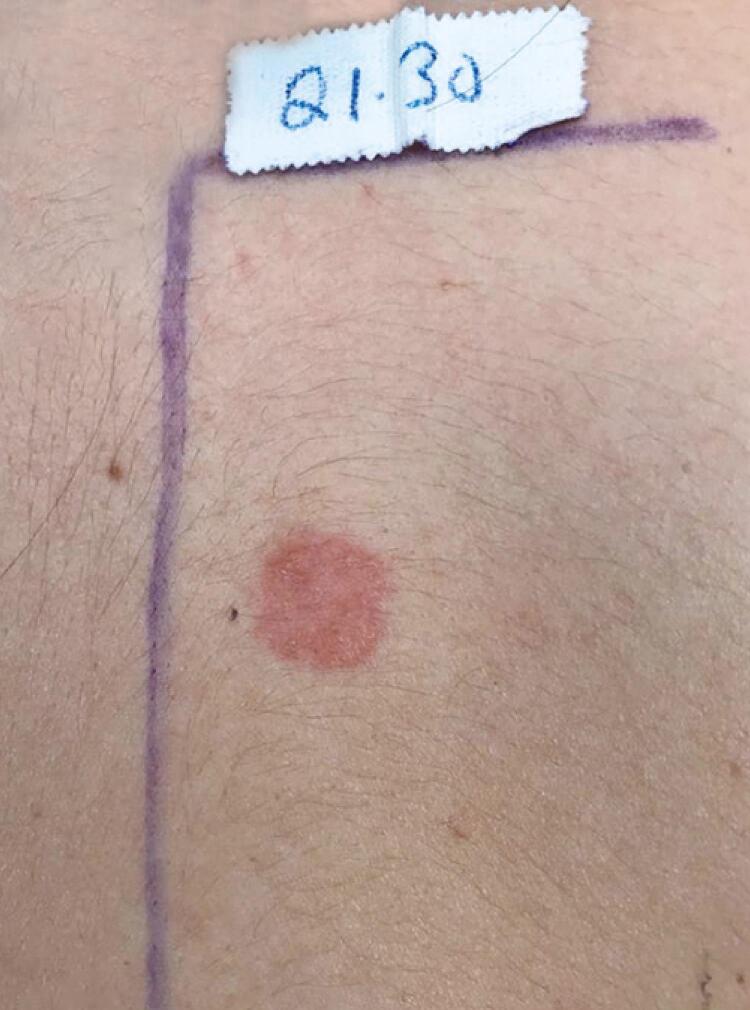
Contact test strongly positive (++) for propolis

Once the clinical diagnosis of allergic contact dermatitis was made, we chose to perform contact tests with a more comprehensive and updated series (Latin American standard series) containing 40 substances. For this, four contact strips - hypoallergenic Alergochamber^®^ (Neoflex Biotecnologia Ltda., Sertãozinho, SP, Brazil) - previously prepared with ten substances each were used. The test substances were manipulated, following their CAS Registry Number^®^, by IPI-ASAC Brasil, according to the orientation of the Ibero-Latin American College of Dermatology (CILAD).^(3)^ Readings were taken at 48 hours (D2) and 96 hours (D4). In the second reading, we observed a strong positive reaction (++) to 10% propolis in petroleum jelly (Figure 3) and a slightly positive reaction (+) to perfume mix I.

This study was approved by the Ethics Committee with (approval number: 3.711.423, CAAE: 22295219.9.0000.5553).

## DISCUSSION

Allergic contact cheilitis result from allergy to chemicals in lip balms and lip glosses, lipsticks, and sunscreens. The anatomy of the lips is surprisingly complex. The lips are frequently exposed to cosmetic products, in which there are dies, flavoring agents, sunscreens, preservatives and other vehicles.^(1)^ However, the young woman denied using such cosmetics or lipsticks and even sunscreens. She used a dexpanthenol-based lip moisturizer, but it was not considered a cause, since its use was more reactive to the worsening of the condition than something that triggered it.

Propolis is a lipophilic, resinous, brownish substance collected by bees from living plants. It is mixed with wax and used for construction and fitting of their hives.^(4)^ It has long been known as an occupational contact allergen in beekeepers. However, most cases of allergy are not occupational. Due to its pharmacological properties, this component is widely used in folk medicine and in the biocosmetic industry. Thus, it is believed the increase in cases of contact dermatitis to propolis seen over the past two decades is probably due to its use in cosmetic and pharmaceutical preparations.^(5)^ Therefore, because of its relevance, in 2019, the standard European contact test series was changed, allowing its inclusion at a concentration of 10% in petroleum jelly.^(6,7)^

Overall, positivity of the reactions has reached 1.9% to 3.5%, according to the literature.^(8)^ In Brazil, there are no data on the prevalence of propolis allergy. Likewise, the substance is not included in the standard^(9)^ or cosmetic series,^(10)^ or in any other specific series. Only the use of a more comprehensive series, supplemented with 10% propolis, has made it possible to diagnose the etiology of eczema. The Latin American series was proposed by CILAD and represents a significant update of the national standard series.^(3)^

One study tested 3,221 patients for propolis, with positive reactions in 112 (3.5%). They also found a cross-reactivity rate of 13% for colophonium and an even higher rate for perfume mix I of 25%. Based on these data, the authors recommended propolis allergic patients should be advised to avoid colophonium and fragrances.^(11)^ The patient also showed reactivity to perfume mix I, although less intense. She was negative for colophonium. Thus, she was advised to read labels and avoid personal use products containing fragrances.

When using the so-called natural cosmetics, individuals tend to underestimate the risk of developing allergic contact dermatitis.^(2)^ This is a common misconception, since such products may contain allergens, such as propolis.^(1)^ Cheilitis is a common presentation of propolis allergy, given the frequency of its use in lip balms or lipsticks.^(2)^ Confronted with the result and the fact she emphatically denied using medications, the patient admitted she believed propolis was not a medicine for being natural, and it would not trigger an allergy. She reported she had been using propolis as oral drops for at least 10 years and, because of the pandemic, she decided to increase the dose to improve immunity. It is interesting to note she used propolis before going to bed, which could explain, in part, the neck involvement, since, due to her past history of rhinitis, she had been mouth breathing, salivating on the pillow. After discontinuing the use of the substance, there was remission of the clinical picture.

## CONCLUSION

This report alerts the medical community to the fact that propolis allergy may be increasing in the general population due to the popularity of natural products. Contact allergy is common in patients with eczematous cheilitis, and contact testing is essential. However, the substance is not present in the Brazilian standard series, which therefore needs to be expanded and updated.
